# The Use of Corticosteroid Randomisation after Significant Head Injury (CRASH) Prognostic Model as Mortality Predictor of Traumatic Brain Injury Patients Underwent Surgery in Low-Middle Income Countries

**DOI:** 10.1155/2024/5241605

**Published:** 2024-06-21

**Authors:** Radian A. Halimi, Iwan Fuadi, Dionisius Alby

**Affiliations:** Department of Anesthesiology and Intensive Therapy, Faculty of Medicine Padjadjaran University/Hasan Sadikin Central General Hospital, Bandung, West Java, Indonesia

## Abstract

**Background:**

Traumatic brain injury (TBI) is a disruption to normal brain functions caused by traumas such as collisions, blows, or penetrating injuries. There are factors affecting patient outcomes that also have a predictive value. Limited data from low-middle income countries showed a high number of poor outcomes in TBI patients. The corticosteroid randomisation after significant head injury (CRASH) prognostic model is a predictive model that uses such factors and is often used in developed countries. The model has an excellent discriminative ability. However, there is still a lack of studies on its use in surgical patients in low-middle income countries. This study aimed to evaluate the CRASH model's validity to predict 14-day mortality of TBI patients who underwent surgery in low-middle income countries.

**Methods:**

This retrospective analytical observational study employed total sampling including all TBI patients who underwent surgery with general anesthesia from January to December 2022. Statistical analysis was performed by applying Mann–Whitney and Fisher exact tests, while the model's discriminative ability was determined through the area under the curve (AUC) calculations.

**Results:**

112 TBI patients were admitted during the study period, and 74 patients were included. Independent statistical analysis showed that 14-day mortality risk, age, Glasgow Coma Scale score, TBI severity, pupillary response, and major extracranial trauma had a significant individual correlation with patients' actual mortality outcome (*p* < 0.05). The AUC analysis revealed an excellent mortality prediction (AUC 0.838; CI 95%).

**Conclusion:**

The CRASH prognostic model performs well in predicting the 14-day mortality of TBI patients who underwent surgery in low-middle income countries.

## 1. Introduction

Traumatic brain injury (TBI) is any disruption of brain normal function due to traumatic mechanisms such as bumps, blows, or penetrating head injury [1]. In low- and middle-income countries, the burden emerges from TBI being greater [2–4]. TBI patients have a high mortality rate [5]. As the long-term outcome, TBI survivors also have a high morbidity rate. Limited data from low- and middle-income countries showed a high number of poor outcomes in TBI patients [2].

Scoring and prognostic systems to predict the outcome of TBI patients can be the cornerstone for effective decision-making and resource allocation [6]. Age, sex, traumatic mechanism, GCS score, pupillary reactivity at admission, and major extracranial injury have been studied as the prognostic factors for the prediction of TBI patients' outcomes [7]. The corticosteroid randomisation after significant head injury (CRASH) trial combines various prognostic factor variables into a prognostic model with a web-based calculator to obtain the TBI patients' mortality rate at 14 days [7, 8].

Previous studies showed that the CRASH prognostic model has good discriminative ability [7, 9]. The study conducted in Indonesia by Faried et al. also proves excellent ability of the CRASH prognostic model when similarly performed in general TBI patients [10]. However, in low- to middle-income countries, various considerations need to be taken into account before deciding on surgery due to resource constraints. Therefore, a good prognostic scoring model should also provide an estimation of the outcome if TBI patients underwent surgery. The study to validate the CRASH prognostic model in TBI patients' subpopulation who underwent surgery was limited especially in low-middle income countries. Due to these reasons, further evaluation on discriminative ability of the CRASH prognostic model was needed in TBI patients who underwent surgery. Therefore, this study aims to investigate the validity of the CRASH prognostic model to predict 14-day mortality of TBI patients who underwent surgery in low-middle income countries.

## 2. Methods

This study used retrospective observational analytics as its research method. Data were collected retrospectively on traumatic brain injury patients who underwent surgery with general anesthesia in the central surgery installation of Hasan Sadikin Central General Hospital, Bandung, from 1st January to 31st December 2022. Patients with incomplete medical record data and pediatric patients under 18 years were excluded in this study subjects. Sampling was conducted using the total sampling method. The data collected were secondary data obtained through research instruments in the form of medical records. Data from the medical record review were statistically compared and analyzed.

This study was conducted after obtaining research approval from the Ethics and Research Committee of Hasan Sadikin Central General Hospital on 6th March 2023 with IRB number: LB.02.01/X.6.5/81/2023.

The independent variables in this study were age, Glasgow Coma Scale (GCS) score, TBI severity, pupillary response, major extracranial trauma, and 14-day mortality risk. Age was assessed in years when the patient arrived at the emergency department (ED), the patient's GCS score was the result of the total GCS score calculation during the initial patient examination in the ED, and the severity of TBI was based on the total GCS score during the initial assessment in the ED divided into three groups: mild (GCS 13–15), moderate (GCS 9–12), and severe (GCS ≤ 8). The pupillary response was categorized into three categories: bilateral, where both eyes exhibited pupil constriction in response to light stimulation; unilateral, where only one eye showed pupil constriction in response to light stimulation; and negative, where both eyes did not exhibit pupil constriction in response to light stimulation. Major extracranial trauma was determined through a physical examination of patients diagnosed with trauma other than TBI requiring hospitalization during the initial assessment. The 14-day mortality risk was the risk of patients dying within 14 days of treatment based on the prognostic CRASH basic model calculation through the website “crash2.lshtm.ac.uk.” The dependent variable in this study was the 14-day mortality of TBI patients who underwent surgery at Hasan Sadikin Central General Hospital, Bandung.

The data were tabulated and analyzed descriptively and statistically and tested for the prognostic model using the AUC discrimination test. Data analysis included univariate and bivariate analyses. The univariate analysis consisted of categorical data in percentages/proportions, while numerical data were presented using means/medians, standard deviation, and minimum and maximum values.

The bivariate analysis was conducted statistically using the chi-square test for the categorical data and the Pearson correlation test for numerical data. As an alternative, the Fisher exact test was conducted for the data that did not meet the criteria for the chi-square test. For the data that were not distributed normally, the analysis was alternatively conducted by the Mann–Whitney test for categorical data and the Spearman test for numerical data. The significance criteria used in this study was *p* value, in which *p* ≤ 0.05 was considered statistically significant. The collected data were recorded on a specific form and processed using the IBM Statistical Product and Service Solution (SPSS) 25.0 for Windows (IBM Corp., Armonk, New York, USA).

The prognostic model discrimination test was conducted using the analysis of the area under receiver operating characteristic (AuROC) curve with the null hypothesis of AuROC = 0.5, which means the prognostic model cannot predict patient mortality. The hypothesis test was considered statistically significant if the *p* value was <0.05 at a 95% confidence level. Sensitivity and specificity tests were conducted based on the optimal cutoff value obtained from the Youden index.

## 3. Results

The data collection yielded 112 adult patients with TBI who underwent surgery at Hasan Sadikin Central General Hospital from January to December 2022. A total of 74 medical records met the inclusion criteria and 38 medical records were excluded due to incomplete information and the patient's age being less than 18 years. The subjects of this study consisted of 63 patients who survived and 11 patients who deceased within 14 days of treatment. The extracted data from medical records were admission and discharge summaries, triage assessments, preoperative assessments, preanesthetic assessments, and anesthesia records.

Based on Table 1, the TBI patients who underwent surgery had a median age of 29 years with an age range of 17 to 71 years in the survived group and a median age of 49 years with an age range of 20 to 69 years in the deceased group. In this study, the majority of subjects were male, both in the survived group (*n* = 50 (79.4%)) and in the deceased group (*n* = 9 (81.8%)). The GCS score in the survived group had a median of 12, with the lowest and highest scores being 5 and 14, respectively, while the median GCS score in the deceased group was 9, with the lowest and highest GCS scores being 4 and 14, respectively. In the survived group, most patients had a mild severity level (47.6%), while there were more patients with a severe severity level (45.5%) in the deceased group. The proportion of patients with bilateral pupil responses in the survived group (96.8%) was greater compared to the deceased group (63.6%). There were 5 patients (7.9%) with major extracranial injury in the survived group, while there were 7 patients (63.6%) in the deceased group.

In this study, data distribution for each variable was not normal. Therefore, we conducted a comparative test between TBI patients who survived and those who died using alternative testing methods. The numerical variables, namely, age and GCS score, were assessed using the Mann–Whitney test. There were statistically significant differences in the proportions of age (*p*=0.012) and GCS score (*p*=0.004) variables between the two groups. The variables of sex, TBI severity, pupillary response, and major extracranial injury were analyzed using the Fisher exact test. There were statistically significant proportion differences for TBI severity (*p*=0.003), pupillary response (*p*=0.0001), and major extracranial injury (*p*=0.0001), except for sex (*p*=0.854).

We calculated the variable of 14-day mortality risk based on the basic CRASH prognostic model through the website crash2.lshtm.ac.uk. In this study, the data were presented using a median since the 14-day mortality risks in both groups were not distributed normally based on the result of normality testing. Based on Table 2, the median of 14-day mortality risk in survived patients was 4, with the lowest and the highest score being 2 and 33, respectively. In the deceased group, the median of the 14-day mortality risk was 32.8%, with the lowest score of 3% and the highest score of 58%. The statistical analysis results using the Mann–Whitney test showed a statistically significant difference (*p*=0.0001), thus concluding a relationship between the 14-day mortality risk and the 14-day mortality of TBI patients who underwent surgery.

The correlation test results using the Spearman test in Table 3 showed a statistically significant correlation for both age (*p*=0.017) and GCS score (*p*=0.003). The age variable had a weak degree of correlation (*r* = 0.276), while the degree of correlation of GCS score was approaching perfect (*r* = 1).

Table 4 presents the result of the Fisher exact test. There was no statistically significant association (*p*=0.852) between sex and 14-day mortality of TBI patients who underwent surgery. In contrast, there were statistically significant associations for the variables of TBI severity (*p*=0.003), pupillary response (*p*=0.0001), and major extracranial injury (*p*=0.024).

**Figure 1:**
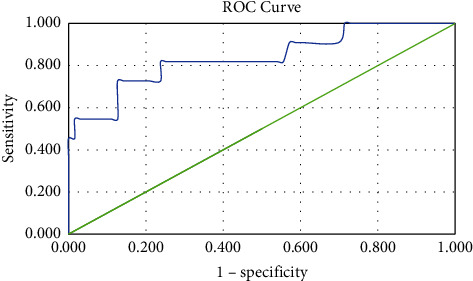
Receiver operating characteristic (ROC) curve analysis of the CRASH prognostic model in determining 14‐day mortality risk of TBI patients.

The discriminative ability analysis of the CRASH prognostic model in determining the 14-day mortality risk of TBI patients who underwent surgery was assessed using the AuROC. In this study, the area under the curve (AUC) analysis results showed a value of 0.838 (95% CI 0.693–0.984), suggesting that the prognostic model can predict patient mortality, as demonstrated in Figure 1 and Table 5. Subsequent analysis using the Youden index to find the maximum cutoff point from the AUC resulted in a cutoff point 15.5, with a sensitivity of 72.7% and specificity of 87.3%.

## 4. Discussion

The prognostic evaluation in patients with TBI is a cornerstone in the decision-making process and the provision of information for patients' relatives. This study aims to assess the discriminative ability of the CRASH prognostic model in patients diagnosed with TBI who underwent surgery. The 14-day mortality risk calculation used a website-based calculator considering several variables, namely, age, GCS score, TBI severity, pupillary response, and major extracranial injury on admission at ED [8, 11]. An additional variable of head CT scan can also support the CRASH prognostic model. This study involved adult TBI patients with a GCS score of 14 or less, as in the original study cohorts [8]. The selection of patients who underwent surgery was carried out to determine the predictive ability of the CRASH score in this subgroup. In our study, it was obtained that sex did not have a statistically significant correlation with the 14-day mortality of TBI patients. This finding accords with the previous meta-analysis, which stated that sex does not influence the outcomes of TBI patients. Thus, sex is not included as a variable in the CRASH prognostic model [6]. Previous studies showed that males have higher vulnerability and proportion for TBI. This is consistent with the finding of this study, which indicated that males dominate both groups [6, 8, 10, 12–15].

Compared to high-income countries, patients from low- and middle-income countries had worse early prognoses and higher 14-day mortality [8]. In low- and middle-income countries, the influence of age on patient outcomes was lower than that of GCS score [8]. The CRASH prognostic model considers the patient's country of origin based on several external validation research studies in several countries [8]. Previous study about using the CRASH prognostic model in Indonesia had shown an excellent discriminative ability to overall TBI patients [10]. In contrast, another study that treated TBI patients using intracranial pressure-targeted therapy showed an overestimation of worse outcomes [16]. To determine the CRASH prognostic model's capability within the population of TBI patients receiving intervention in general anesthesia, we conducted a study with TBI patients who underwent surgery as the subjects.

Patients' age is one of the basic variables in the CRASH prognostic model. This study found a statistically significant difference between patients' age and mortality. The group of deceased TBI patients had an age median of 49 years, consistent with the previous study that showed the linear correlation between the occurrence of TBI after the age of 40 years and poorer outcomes [8]. This is related to extracranial comorbidities, changes in brain plasticity, or differences in clinical management to different ages [8]. Older age is also an independent predictor of higher mortality risk after improving the GCS score. Increasing age is correlated to higher mortality in every GCS score [8]. However, in patients younger than 55 years, GCS score is known to be a better predictor.

GCS score is the key predictor in evaluating TBI patients' prognosis. GCS score is also used in determining the severity of TBI patients. In this study, most TBI patients had mild severity, which aligns with the previous study [6]. The degree of patient outcomes is known to be linearly correlated with patient's GCS score [8]. This study yielded a statistically significant result on its correlative test that showed a nearly perfect (*r* = 1) correlation between GCS score and 14-day mortality. Nonetheless, 2 patients with a good initial GCS score (GCS 13-14) in this study died within 14 days of hospitalization. A medical record review was conducted for both patients. The first patient was known to have undergone surgery on the first day of hospitalization and had deterioration of GCS score as well as hemodynamic instability. The other patient was suspected of aspiration on the sixth postoperative day of hospitalization due to trauma to the facial area.

In this study, the comparative test showed a statistically significant difference in pupillary response of TBI patients between survived and deceased groups. A previous study found that pupillary response was the third most robust predictor of 14-day mortality outcomes [8]. TBI patients with negative pupil response showed a higher mortality rate than patients with positive pupil response [8]. The loss of pupillary response indicates the dysfunction of the brainstem, which is responsible for several essential functions [17]. Moreover, acute findings of abnormal pupil after TBI may occur due to the compression of the third cranial nerve and subsequent brainstem, uncal or transtentorial herniation in the temporomedial lobe, or reduced blood flow to the brainstem [6].

In addition to those variables, the CRASH prognostic model includes major extracranial injury as a variable in estimating the 14-day mortality risk of TBI patients. Major extracranial injury was also the predictor of TBI patients' mortality in the previous study [10]. In this study, there was a statistically significant difference between the presence and absence of major extracranial injury and the 14-day mortality of TBI patients who underwent surgery. This is in line with the findings of the previous study regarding the presence of comorbidities in major extracranial injury, namely, hypotension, hypoxia, coagulopathy, and worse injury, patients [18].

After independently analyzing the correlation between each of the variables in the CRASH prognostic model and the mortality of TBI patients who underwent surgery, the calculated results of 14-day mortality risk based on the CRASH prognostic model through the website crash2.lshtm.ac.uk were compared to the actual mortality of the subjects. This study obtained a significant relationship between 14-day mortality risk and actual mortality of TBI patients who underwent surgery with general anesthesia in Hasan Sadikin Central General Hospital.

The discriminative ability of the CRASH prognostic model was assessed using the AUC calculation method. The value obtained from this basic model analysis was 0.838 with 95% CI (0.693–0.984), indicating a good level of mortality determination for this prognostic model. This result accords with the initial study, which showed the AUC ability of the CRASH prognostic model in the range of 0.82–0.88 [8, 12]. This AUC result is lower than the previous study in Indonesia in which the AUC value was 0.98 [10]. This difference was perchance caused by the smaller sample size obtained in this study and the usage of the basic CRASH prognostic model without the utilization of CT scan findings. Previous study from other low- and middle-income countries also showed a lower AUC value with the range of 0.64–0.73 [7].

The optimal cutoff point obtained in this study was 15.5 based on the Youden index calculation, with sensitivity and specificity being 72.7% and 87.3%, respectively. Therefore, it can be inferred that 83.8% (AUC 0.838) of TBI patients who underwent surgery with a 14-day mortality risk of more than 15.5% have a higher chance to decease in 14 days of hospitalization, particularly in Hasan Sadikin Central General Hospital. The different results regarding optimal cutoff point, sensitivity, and specificity compared to previous study perchance due to the wide range of the 14-day mortality risk as well as the non-normally distributed data in this study [10].

Predicted mortality based on the CRASH prognostic model can be used to assist in the decision-making process as well as providing information to patients' relatives. However, it should be emphasized that other factors still influence patient outcomes. Hence, this prognostic model cannot replace clinical consideration outright.

This study has several limitations, including not encompassing the CT scan findings for calculating the CRASH prognostic model. This limitation arises from the limited availability of CT scan data in the patient's medical records. Opting for a complete case analysis while excluding incomplete medical record data may also result in information loss in this study.

This study only assessed the predictive ability of the CRASH prognostic model for 14-day mortality and did not include the predictive ability for the 6-month outcomes of TBI patients. The results of this study only calibrate to the CRASH model's overall predictive ability in TBI patients who underwent surgery. Further study with a larger population, including TBI patients who did not undergo surgery, is needed to perform subgroup comparison measurements and prevent overestimation of the CRASH model's performance in this population.

This study concludes that there is a significant relationship between the 14-day mortality risk, age, GCS score, TBI severity, pupillary response, and major extracranial injury with the actual mortality outcome of TBI patients who underwent surgery with general anesthesia in Hasan Sadikin Central General Hospital. The CRASH prognostic assessment demonstrates an excellent predictive ability for the 14-day mortality of TBI patients who underwent surgery in Hasan Sadikin Central General Hospital. Based on this study, the CRASH prognostic assessment can be utilized to predict the 14-day mortality of TBI patients who underwent surgery in low-middle income countries.

## Figures and Tables

**Table 1:** Characteristics of study subjects.

Variables	TBI patients		*P* value^†^
	Survived *n* = 63	Deceased *n* = 11	
Age			
Median	29	49	0.012^*∗*^
Range (min-max)	17−71	20−69	
Sex			
Male	50 (79.4%)	9 (81.8%)	0.854
Female	13 (20.6%)	2 (18.2%)	
GCS score			
Median	12	9	0.004^*∗*^
Range (min-max)	5−14	4−14	
TBI severity			
Mild	30 (47.6%)	2 (18.2%)	0.003^*∗∗*^
Moderate	28 (44.4%)	4 (36.4%)	
Severe	5 (7.9%)	5 (45.5%)	
Pupillary response			
Bilateral	61 (96.8%)	7 (63.6%)	0.0001^*∗∗*^
Unilateral	2 (3.2%)	2 (18.2%)	
Negative	0 (0%)	2(18.2%)	
Major extracranial injury			
Present	5 (7.9%)	58 (92.1%)	0.0001^*∗∗*^
Absent	58 (92.1%)	4 (36.4%)	

Note. GCS: Glasgow Coma Scale and TBI: traumatic brain injury. ^†^The *P* value is calculated using nonparametric statistic test, ^*∗*^the Mann–Whitney test significance of *α* = 0.05, and ^*∗∗*^the Fisher exact test significance of *α* = 0.05.

**Table 2:** Comparison of 14-day mortality risk in TBI patients who underwent surgery in survived and deceased groups.

Variables	TBI patients		*P* value
	Survived (*n* = 63)	Deceased (*n* = 11)	
14-day mortality risk			
Median	4	2−33	0.0001^*∗*^
Range (min-max)	32.8	3–58	

Note. TBI: traumatic brain injury. ^*∗*^The *P* value is calculated using the Mann–Whitney test significance of *α* = 0.05.

**Table 3:** Correlation of age and GCS score to the 14-day mortality of TBI patients who underwent surgery.

Correlation	*r*	*P* value	*N*
Age	0.276	0.017^*∗*^	74
GCS score	1	0.003^*∗*^	74

Note. GCS: Glasgow Coma Scale. ^*∗*^The significance value of the Spearman test is based on a *P* value <0.05, meaning it is statistically significant.

**Table 4:** Comparison of TBI patients who underwent surgery in survived and deceased groups.

Variables	TBI patients				Total		OR (95% CI)	*P* value
	Survived		Deceased		*n*	%		
	*n*	%	*n*	%				
Sex								
Male	50	84.7	9	15.3	59	100	0.855	0.852
Female	13	86.7	2	13.3	15	100		
TBI severity								
Mild	30	93.8	2	6.3	32	100	9.274	0.003^*∗*^
Moderate	28	87.5	4	12.5	32	100		
Severe	5	50	5	50	10	100		
Pupillary response								
Unilateral	2	50	2	50	4	100	11.284	0.0001^*∗*^
Bilateral	61	89.7	7	10.3	68	100		
Negative	0	0	2	100	2	100		
Major extracranial injury								
Present	5	55.6	4	44.4	9	100	6.629	0.024^*∗*^
Absent	58	89.2	7	10.8	65	100		

Note. OR: odds ratio and TBI: traumatic brain injury. ^*∗*^The Fisher exact test significance of *α* = 0.05.

**Table 5:** The AUC analysis results of 14-day mortality risk of TBI patients.

	AUC	Std. error	*P* value	95% CI	Cutoff point	Sensitivity (%)	Specificity (%)
14-day mortality risk	0.838	0.074	0.0001^*∗*^	0.693–0.984	15.5	72.7	87.3

Note. AUC: area under the curve. ^*∗*^Analysis of AUC using significance *P* value <0.05.

## Data Availability

The patient medical records' data used to support the findings of this study are restricted by the Ethics and Research Committee of Hasan Sadikin Central General Hospital to protect patient's privacy. Data are available from the corresponding author (Dionisius Alby; dionisius19001@mail.unpad.ac.id) for researchers who meet the criteria for access to confidential data.

## References

[1] Capizzi A., Woo J., Verduzco-Gutierrez M. (2020). Traumatic brain injury: an overview of epidemiology, pathophysiology, and medical management. *Medical Clinics of North America*.

[2] Samanamalee S., Sigera P. C., De Silva A. P. (2018). Traumatic brain injury (TBI) outcomes in an LMIC tertiary care centre and performance of trauma scores. *BMC Anesthesiology*.

[3] Dewan M. C., Rattani A., Gupta S. (2019). Estimating the global incidence of traumatic brain injury. *Journal of Neurosurgery*.

[4] Adil S. M., Elahi C., Patel D. N. (2022). Deep learning to predict traumatic brain injury outcomes in the low-resource setting. *World Neurosurgery*.

[5] Agrawal D., Ahmed S., Khan S., Gupta D., Sinha S., Satyarthee G. (2016). Outcome in 2068 patients of head injury: experience at a level 1 trauma centre in India. *Asian Journal of Neurosurgery*.

[6] Raj R. (2015). Prognostic models in traumatic brain injury. *Acta Anaesthesiologica Scandinavica*.

[7] Wongchareon K., Thompson H. J., Mitchell P. H., Barber J., Temkin N. (2020). IMPACT and CRASH prognostic models for traumatic brain injury: external validation in a South-American cohort. *Injury Prevention*.

[8] Mrc Crash Trial Collaborators, Arango M., Clayton T., Edwards P., Komolafe E., Poccock S. (2008). Predicting outcome after traumatic brain injury: practical prognostic models based on large cohort of international patients. *BMJ*.

[9] Dijkland S. A., Helmrich I. R. R., Nieboer D. (2021). Outcome prediction after moderate and severe traumatic brain injury: external validation of two established prognostic models in 1742 European patients. *Journal of Neurotrauma*.

[10] Faried A., Satriawan F. C., Arifin M. Z. (2018). Feasibility of online traumatic brain injury prognostic corticosteroids randomisation after significant head injury (CRASH) model as a predictor of mortality. *World Neurosurgery*.

[11] Brennan P. M., Murray G. D., Teasdale G. M. (2018). Simplifying the use of prognostic information in traumatic brain injury. Part 1: the GCS-Pupils score: an extended index of clinical severity. *Journal of Neurosurgery*.

[12] Dullaert M., Oerlemans J., De Paepe P., Kalala Okito J. P., Hallaert G. (2020). Comparison of the CRASH Score–predicted and real outcome of traumatic brain injury in a retrospective analysis of 417 patients. *World Neurosurgery*.

[13] Faried A., Bachani A. M., Sendjaja A. N., Hung Y. W., Arifin M. Z. (2017). Characteristics of moderate and severe traumatic brain injury of motorcycle crashes in Bandung, Indonesia. *World Neurosurgery*.

[14] Chandra J., Tobing W. L., Chandra J. (2021). Risk factors of mortality due to traumatic brain injury in Marsidi Judono general hospital Belitung Indonesia. *Indonesian Journal of Neurosurgery*.

[15] Syahrul S., Imran I., Fajri N. (2020). Clinical characteristics of traumatic brain injury patients in dr. Zainoel abidin public hospital banda aceh, Indonesia. *Bali Medical Journal*.

[16] Olivecrona M., Olivecrona Z. (2013). Use of the CRASH study prognosis calculator in patients with severe traumatic brain injury treated with an intracranial pressure-targeted therapy. *Journal of Clinical Neuroscience*.

[17] Ercole A., Menon D. K., Matta B. F., Menon D. K., Smith M. (2011). Traumatic brain injury. *Core Topics in Neuroanaesthesia and Neurointensive Care [Internet]*.

[18] Watanabe T., Kawai Y., Iwamura A., Maegawa N., Fukushima H., Okuchi K. (2018). Outcomes after traumatic brain injury with concomitant severe extracranial injuries. *Neurologia Medico-Chirurgica*.

